# Supplementary material for: Conservation of imprinted expression across genotypes is correlated with consistency of imprinting across endosperm development in maize
Source: G3 (Bethesda). 2025 Feb 14;15(4):jkaf028. doi: 10.1093/g3journal/jkaf028 (PMC12005164; doi:10.1093/g3journal/jkaf028)
Supplement: jkaf028_Supplementary_Data [file jkaf028_supplementary_data.zip › Supplemental_Figure_Legends_G3-2025-405671.docx]

**Supplemental Figure Legends**

**Figure S1.** Kmeans clusters of genes over time. Genes were normalized to rpm at each time point divided by max rpm at any time point. Blue lines represent average normalized expression of each gene across 3 replicates for each time point. Red lines represent the average of all genes in that cluster in B73. (A) Reads for W22xB73 crosses mapped to B73 genome. (B) Zeins in B73xW22 crosses, and W22xB73 crosses. (C) Imprinted genes in groups 1-4 mapped to kmeans clusters in B73. Each colored line is the average expression for an individual gene across three replicates for each time point assessed. Thick black lines are the kmeans of all genes within the group as decided by the original clustering of all genes expressed in B73. Genes are colored by which imprinting group the belong to.

**Figure S2.** Expression patterns compared to maternal preference across lines. Maroon indicates the maternal preference in that cross meets the threshold for RER to be maternally expressed, blue indicates preference meets the RER threshold to be paternally expressed. Purple horizontal line indicates biparental expression average, green horizontal line indicates maternal RER threshold, blue horizontal line indicates paternal RER threshold. Barplots of maternal preference across lines for example genes of MEGs and PEGs in the four time series groups.
