# Supplementary figures and images for: Conservation of imprinted expression across genotypes is correlated with consistency of imprinting across endosperm development in maize
Source: G3 (Bethesda). 2025 Feb 14;15(4):jkaf028. doi: 10.1093/g3journal/jkaf028 (PMC12005164; doi:10.1093/g3journal/jkaf028)

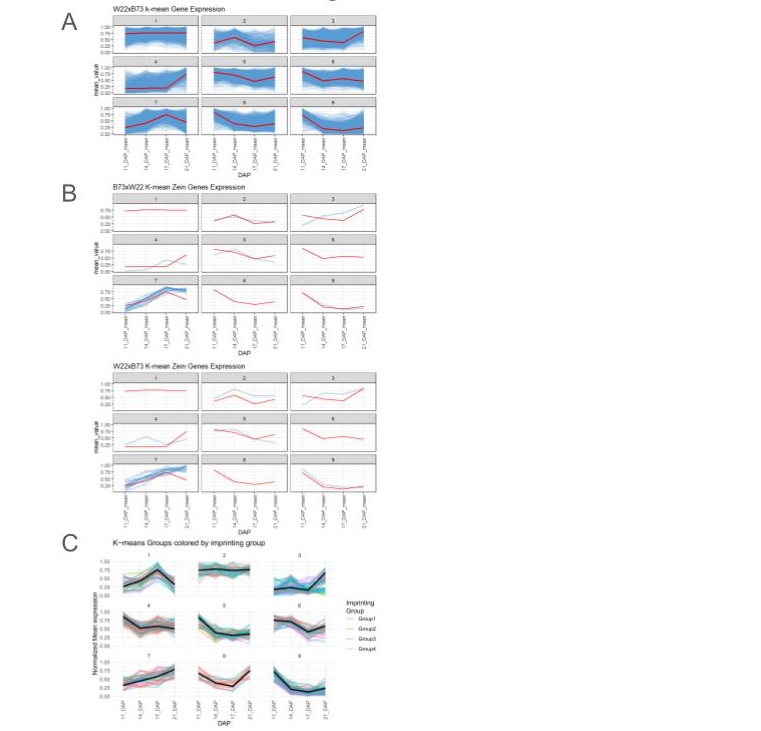

Supplement: jkaf028_Supplementary_Data [file jkaf028_supplementary_data.zip › Figure_S1_G3-2025-405671.jpg]

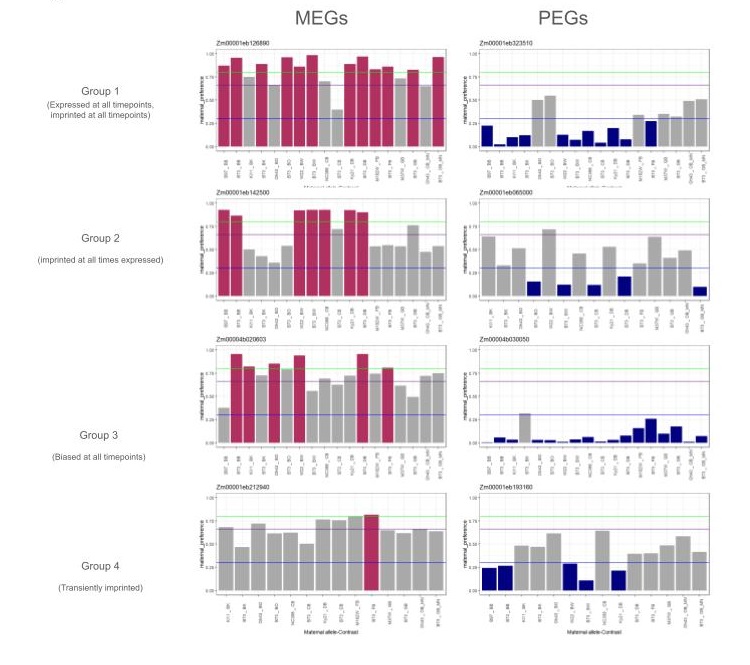

Supplement: jkaf028_Supplementary_Data [file jkaf028_supplementary_data.zip › Figure_S2_G3-2025-405671.jpg]
